# Reviews

**Published:** 1892-02

**Authors:** 


### REVIEWS.

---

The Physicians' Visiting List for 1892. P. Blakiston, Son & Co., Philadelphia.

This little book, now in its forty-first year of its publication, is, as usual, somewhat of an improvement on its predecessors. It contains a list of new remedies, poisons and their antidotes, dose table, &c. As a convenient, ready reference, it has no equal.

The Veterinarians' Call-Book (perpetual) and Yearly Register. By Roscoe R. Bell, D. V. S., Professor of Materia Medica, Therapeutics and Hygiene in the American Veterinary College, New York; President of the Long Island Veterinary Society; U. S. Government Veterinary Inspector, etc., etc. Sabiston & Murray, New York, 1891.

A visiting list which can be commenced at any time and used until full, containing much useful information for the student and the busy practitioner, to which is added an Annual Record, giving a list of all the veterinary societies in the United States and Canada, their officers, times and places of meeting, veterinary colleges and their faculties, alumni associations, and other matter of interest to the profession. Contains a list of the chief veterinary drugs, their uses and doses; a list of poisons and their antidotes, etc., etc. An indispensable pocket book for the veterinarian.

Age of the Domestic Animals. By R. S. Huidekoper, M. D., Veterinarian (Alfort), Professor of Sanitary Medicine and Veterinary Jurisprudence, American Veterinary College, New York; Honorary Associate of the Royal College of Veterinary Surgeons, London; etc., etc.

The older readers of the Journal will recognize in this book the revision of a series of articles on the anatomy of the teeth, dentition and the various means of determining the age of the domestic animals, which appeared through 1890 and 1891. In the revised book form the text has been considerably augmented, and some forty additional illustrations have been added. It is a necessary text-book for students and an authority for the library of the veterinary surgeon and the horseman.

THE DOG IN HEALTH AND DISEASE, including his origin, varieties, breeding, education, and general management in health and his treatment in disease. By Wesley Mills, M. A., M. D., D. V. S., etc., etc., Professor of Physiology in the Faculty of Human Medicine, and in the Faculty of Comparative Medicine and Veterinary Science of McGill University, Montreal; Lecturer on Cynology in the latter faculty, and author of Animal Physiology, Comparative Physiology, etc. With thirty-eight full page cuts, one colored plate, and numerous other illustrations. D. Appleton & Company, New York, 1892.

Dr. Mills gives, in Part I, 175 pages, an account of the origin and history of the dog, its zoological position and anatomy, a classification of the various breeds, and the rules for management of dogs in health, exercise, care, breeding, feeding, etc. Part II, 225 pages, is devoted to the diseases of the dog and their treatment. While a text-book for the veterinarian it is an attractive and readable book for the general public.

THE INTERNAL PARASITES OF THE HORSE (Entozoa). By J. T. Duncan, M. D., C. M., V. S., Lecturer on the Entozoa of the Domesticated Animals in the Ontario Veterinary College; Honorary Associate of the R. C. V. S., London; Member of the Asssociation of American Anatomists, etc. Illustrated. Presbyterian News Co., Toronto, 1891.

It is to be regretted that Dr. Duncan did not enlarge upon his subject, which treats of the general considerations regarding parasites, their classification, and the specific character, habitat, development, diagnosis, and treatment of them in the horse.

*Lectures:* 1. Introductory; 2. Agnosticism; 3. Valedictory; by Prof. Alex. W. Steen, M. D. Holt Brothers, New York. Three lectures of useful advice to students, and of useful instruction to parents and teachers who should guide students.

BOOKS AND PAMPHLETS RECEIVED.

Jahres Bericht der Schlesischen Gesellschaft fur vaterlandische Culture. Breslau, 1891.

Erganzungscheft zum 68 jahresberecht der Schlesichen Gesellchaft fur vaterlandische Culture. Breslau, 1890.

Atti della R. Accademia delle Scienze di Torino. Turin, 1890–91.

Tertiary Fossils of North American Birds. By R. W. Shufeldt, M.D. Rep. from the Auk, Vol VIII., No. 4.

The Canadian Record of Science. Vol. IV., No. 7. Montreal.

Bulletin of the Buffalo Society of Natural Science. Vol. V., No. 3.

Transactions of the Canadian Institute. Vol. II., Part 1, No. 3. Toronto, 1891.

Journal of Proceedings of the Hamilton Association. Part VII. Hamilton, Canada, 1890–91.

Bulletin John Hopkins University, Baltimore.

